# Comment on: What are the evidence bases for developing models of rehabilitation for older people with mental illness in Australia?

**DOI:** 10.1177/10398562231224166

**Published:** 2024-01-09

**Authors:** Nagesh B. Pai, Shae-Leigh C. Vella

**Affiliations:** 8691 Wollongong, NSW; 8691 Wollongong, NSW

Dear Editor,

McKay and Jackson^1^ article highlighted critical issues regarding the care of older people with mental illness (OPMI) in Australia. One issue is the lack of a solid evidence base for the care of OPMI. This is a fundamental issue resulting from a lack of concern and the abandonment of hope for this cohort. This lack of concern and hope is fed by the intersection of both mental illness-related stigma and age-related stigma.

Mental illness-related stigma contributes to people being viewed and treated in a negative manner.^2^ Similarly, age-related stigma impacts upon how an individual is perceived and treated.^2^ OPMI are doubly impacted by the negative views of society.^2^ These views can influence attitudes towards OPMI both consciously and unconsciously.^3,4^ These attitudes in turn can affect the expectations that clinicians have for OPMI.

In Australia, the goal of mental health care (MHC) is meant to be recovery, with a focus upon self-determination and choice. At the centre of recovery is the belief that the individual with mental illness is capable of positive outcomes. The concept of recovery is meant to be central for both adults under and over 65 years of age; however, previous research has indicated that healthcare workers are less likely to engage in recovery-orientated practice with OPMI.^5^ Conversely, previous research has demonstrated that older people’s MHC services do not have recovery as the primary goal.^3,4^ Koder^3^ found that staff held poor attitudes towards recovery including a lack of hope for this cohort. The staff also expressed paternalistic attitudes towards the care of OPMI further diverting from recovery-oriented care.^3^ Koder^3^ concluded that cognitive and physical limitations evident in this group and available services were barriers to recovery-oriented care in this cohort. Similarly, Tabatabaei-Jafari and colleagues^4^ found that there were less services that support recovery for this cohort. Thus, acknowledging and trying to overcome the barriers to recovery-oriented care for OPMI will assist with the provision of recovery-oriented care to OPMI. Such a policy-based shift will support OPMI to enjoy the best possible quality of life.

## References

[1] McKay R Jackson K . What are the evidence bases for developing models of rehabilitation for older people with mental illness in Australia? Australas Psychiatry, 2023; 31(5): 601–606. 37615592 10.1177/10398562231190831 PMC10566217

[2] De Mendonca Lima CA . The reduction of stigma and discrimination against older people with mental disorders: a challenge for the future. Arch Geront Geriatr 2004; 38(Supp): 109–120. 10.1016/j.archger.2004.04.018 15207405

[3] Koder D . Recovery-Oriented care for older people: staff attitudes and practices. The Int J Psychosoc Rehabilitation 2018; 22(1): 46–54.

[4] Tabatabaei-Jafari H Salinas-Perez JA Furst MA , et al. Patterns of service provision in older people’s mental health care in Australia. Int. J.Environ. Res.Public Health 2020; 17(22): 8516. 33212966 10.3390/ijerph17228516 PMC7698522

[5] McKay R Jackson K Stevens J . Implementing recovery-orientated practice in older persons mental health services: the NSW experience. AHR 2022; 46: 426–431. 34809748 10.1071/AH21155

